# 基于多孔石墨碳柱的高效液相色谱法测定电化学合成尿素产物

**DOI:** 10.3724/SP.J.1123.2023.09013

**Published:** 2024-08-08

**Authors:** Rui SHEN, Yongyi LI, Xiaojing GAO, Chaoou SHI

**Affiliations:** 华东理工大学分析测试中心, 上海 200237; Analysis and Measurement Center, East China University of Science and Technology, Shanghai 200237, China

**Keywords:** 多孔石墨碳柱, 高效液相色谱, 尿素, 阴离子, porous graphitic carbon column, high performance liquid chromatography (HPLC), urea, anions

## Abstract

电化学合成尿素法是一种相对绿色环保的尿素制备方法,其反应产物中,除目标产物尿素外,还含有大量 $N{O_{3}}^{-}$ 、 $N{O_{2}}^{-}$ 、 $C{O_{3}}^{2-}$ 等杂质离子,这些离子易对尿素测定产生干扰。本文基于多孔石墨碳柱特殊的极性保留效应,并结合其柱流失远小于普通反相C18色谱柱、在极低紫外吸收波长下基线稳定的优势,利用多孔石墨碳柱建立了电合成尿素产物中尿素的高效液相色谱分析方法。经过色谱条件优化后,选择Hypercarb^TM^多孔石墨碳柱(100 mm×4.6 mm, 5 μm),采用水-25 mmol/L甲基磺酸溶液为流动相进行梯度洗脱分离,柱温为30 ℃,流速为1.0 mL/min,进样量为25.0 μL,检测波长190 nm,可实现不受电解液中其他杂质(如 $N{O_{3}}^{-}$ 、 $N{O_{2}}^{-}$ 、 $C{O_{3}}^{2-}$ 等离子)干扰的尿素测定。结果表明,单次样品分析可在15 min内完成,尿素在0.1~100 mg/L范围内线性关系良好,相关系数(*r*^2^)不小于0.9988;方法的检出限(*S/N*=3)和定量限(*S/N*=10)分别为0.028 mg/L和0.093 mg/L,低、中、高3个水平下的加标回收率为112.0%~118.4%。采用所建立的方法对实际电化学合成尿素反应液样品进样分析,结果显示尿素色谱峰的峰形较为良好。该方法前处理简单,方便快捷,尿素与其他干扰离子实现了一定程度的分离,结果准确可靠,特异性好,可用于实际电合成尿素电解液产物中微量尿素及其他相关离子的检测。

## Abstract (en)

Urea is a simple organic compound that is widely used in both the industry and daily life. Compared with conventional methods, the preparation of urea by electrochemical synthesis is more environmentally friendly and sustainable. However, after the reaction, low amounts of urea and high concentrations of inorganic ions, including $N{O_{3}}^{-}$ , $N{O_{2}}^{-}$ , $N{H_{4}}^{+}$ , $H{{\mathrm{CO}}_{3}}^{-}$ , and $C{O_{3}}^{2-}$ , with a comparatively high concentration of $N{O_{3}}^{-}$ , remain in the solution. These ions tend to interfere with the urea measurements. Thus, detecting traces of urea in highly concentrated ionic matrices is challenging. Several urea detection methods, such as infrared spectrometry, the urease method, and high performance liquid chromatography (HPLC), are available. Among these methods, HPLC shows greater sensitivity and accuracy. However, urea is difficult to separate from $N{O_{3}}^{-}$ on reversed-phase C18 columns. Porous graphitic carbon (PGC) columns have lower column loss and better baseline stability at low UV absorption wavelengths than ordinary reversed-phase C18 columns. In this study, a qualitative and quantitative analytical method based on a PGC column was established to detect urea from inorganic ions. The separation of urea from other ions was successfully achieved on a Hypercarb^TM^ PGC column (100 mm×4.6 mm, 5 μm). Experimental investigations were performed under the optimal chromatographic conditions, and gradient elution was performed with a H_2_O-25 mmol/L methanesulfonic acid aqueous solution as the mobile phase (initial mobile phase volume ratio, 98∶2). The column temperature was 30 ℃, the flow rate was 1.0 mL/min, and the injection volume was 25.0 μL. A diode array detector with a detection wavelength of 190 nm was selected because of the low UV absorption wavelengths of urea and impurity ions. The electrolyte product was passed through a 0.22 μm aqueous-phase filter membrane, and the resulting filtrate was analyzed under the optimized conditions. The results showed that urea was well separated from the other ions in the filtrate within 15 min. The urea measurements were unaffected by other ions present in the electrolyte. Moreover, none of the retention times of potentially interfering ions overlapped with those of urea. The background noise remained low and the baseline was smooth, even at a low detection wavelength of 190 nm. Methodological verification experiments showed that urea had a good linear relationship in the mass concentration range of 0.1-100 mg/L (*r*^2^≥0.9988). The limits of detection and quantification were 0.028 and 0.093 mg/L, respectively. When the electrolyte product was spiked with urea at three levels, the average recoveries were in the range of 112.0%-118.4%. Finally, an actual electrolyte sample was analyzed, and the results showed that urea could be detected quantitatively in this sample. The established method requires fewer pretreatment procedures because the samples only need to be filtered and analyzed. It also has a short analysis time and yields accurate, reliable, and specific results. Moreover, at an ultralow detection wavelength of 190 nm, the method demonstrated high sensitivity to urea and ensured a low background noise and baseline stability. Owing to the specific retention effect of the PGC column, the separation of urea at a high $N{O_{3}}^{-}$ concentration was achieved without interference. Thus, the developed method can be applied for the detection of trace urea and other related ions in urea-containing electrolyte products.

尿素(urea),又称脲或碳酰胺,是最简单的有机化合物之一,其化学性质并不稳定,在酸、碱或酶的作用下易水解,在高温下易发生缩合反应。尿素广泛存在于自然界中,是哺乳动物和两栖动物氮排泄的主要代谢产物,还可作为农作物肥料、动物饲料、日用品、炸药等产品的原料;也是许多日常护肤产品的主要成分,可用于临床中各种皮肤病的治疗^[1]^,与日常生活息息相关。

电化学合成尿素法可以在一定程度上弥补传统尿素制备所带来的能源消耗和环境污染问题^[2]^。该方法主要是以N_2_或其他氮源和CO_2_作为原料,在水中电耦合生成尿素^[3]^。由于亚硝酸盐的解离能更低,在合成反应中有利于节约成本、降低能耗^[4]^,可采用碳酸氢根和硝酸盐或亚硝酸盐电解液与CO_2_一同反应进行制备^[5]^。在电化学合成法反应液中,鉴于方法产率较低,除含有少量产物尿素外,还存在大量 $N{O_{3}}^{-}$ 、 $N{O_{2}}^{-}$ 、 $C{O_{3}}^{2-}$ 等杂质离子,易对尿素测定产生干扰。

目前国内外关于尿素(特别是微量及痕量尿素)的检测方法已有多项报道,主要有脲酶法^[6⇓-8]^、比色法^[9,10]^、红外光谱法^[11]^及高效液相色谱法(HPLC)^[12,13]^等。刘灵辉等^[7]^采用改进的脲酶-谷氨酸脱氢酶偶联法可实现游泳池水中尿素含量的自动化分析;Chen等^[9]^和付智慧^[10]^分别选取二乙酰一肟和对二甲氨基苯甲醛为显色剂,使其与尿素发生显色反应后,利用分光光度计进行测定;谭良锋等^[14]^采用中红外光谱法进行车用尿素的定量分析。其中,脲酶法和比色法需要对样品进行复杂的预处理操作,灵敏度和精确度有所损失;红外光谱法一般可直接进样,方便快捷,但灵敏度相对较低;HPLC具有检测快速、便捷灵敏等优点,由于尿素属于强极性化合物,难以在传统液相色谱柱上显著保留,常采用亲水作用色谱法^[15]^、柱前衍生-荧光检测法^[12,16]^、串联质谱法^[17]^等来提高检测灵敏度。其中,Zhang等^[12]^采用荧光检测器对酒精饮料中的尿素进行测定,检出限为0.003 mg/L; Kramer等^[17]^对比了串联质谱和荧光检测两种方法测定饲料中的尿素含量,检出限分别为3 mg/kg和2 mg/kg。

尿素的紫外吸收波长在190 nm左右,处于远紫外吸收区,因此在使用紫外检测器时,检测易受到干扰。多孔石墨化碳(porous graphitic carbon, PGC)柱作为一种于20世纪80年代开始商品化的色谱柱,在色谱分析方面有着优于反相键合硅胶柱的一些独特性能。填料PGC是一种层状交织的特殊导电材料,石墨层的交织特性使其具有高度稳定性和机械强度,耐强酸强碱,耐高温,非常适用于严苛实验条件下的物质分离^[18]^。同时,特殊的石墨极性保留效应使其在极性物质^[19⇓-21]^的分离上有着极大的优势,将PGC柱用于电解液体系下尿素的分离,并在190 nm低紫外波长下进行检测在理论上是可行的。

国内外已有文献报道了电合成尿素反应液中微量尿素的分析方法。刘洋等^[22]^和Meng等^[4]^均采用NH_2_ Phenomenex色谱柱,分别以乙腈水溶液和甲醇水溶液为流动相进行电解液中尿素含量的测定,但还未有利用PGC柱进行分离检测的相关方法报道。由于在对电化学合成尿素产物的检测过程中容易存在基体浓度高、离子干扰多等问题,在传统C18柱上难以将尿素与 $N{O_{3}}^{-}$ 等离子进行分离,本研究利用PGC柱特殊的保留效应,探讨其应用于HPLC并进行电解液体系下尿素检测的可行性。通过对色谱条件的优化,实现了实际电化学合成尿素电解反应液中尿素的分离和检测。

## 1 实验部分

### 1.1 仪器、试剂与材料

U3000高效液相色谱仪(美国Thermo公司),配二极管阵列检测器;AL204电子天平(梅特勒-托利多公司); Millipore-Q A10超纯水机(美国Merck Millipore公司); 100~1000 μL移液枪(北京大龙公司)。硫酸(96%,国药集团化学试剂有限公司);磷酸(85%水溶液)、甲基磺酸(MSA, 99.5%)、尿素(99.5%,生物分子级)、无水碳酸钠(优级纯)、无水碳酸氢钠(优级纯)、氢氧化钠(优级纯)均购自上海阿拉丁生化科技公司;水中 $N{O_{3}}^{-}$ 、 $N{O_{2}}^{-}$ 、 $N{H_{4}}^{+}$ 单一标准溶液,质量浓度均为100 mg/L,上海市计量测试技术研究院。

### 1.2 溶液配制及样品前处理

#### 1.2.1 标准溶液的配制

尿素标准溶液:准确称量100.5 mg尿素至100 mL容量瓶中并用超纯水定容,得到1000 mg/L的尿素储备液,备用;根据情况分别取适量标准储备液,用超纯水稀释成不同浓度的标准溶液。

模拟电解液溶液:准确称取1.011 g硝酸钾和1.060 g碳酸钠至100 mL容量瓶中并用超纯水定容,得到0.1 mol/L电解液,备用。

模拟电解液尿素样品溶液:准确称取1.011 g硝酸钾、1.060 g碳酸钠和100.5 mg尿素至100 mL容量瓶中并用超纯水定容,再利用0.1 mol/L电解液逐级稀释得到0.1 mol/L电解液背景下的系列浓度尿素样品溶液。

#### 1.2.2 样品前处理方法

电合成尿素的实际样品溶液以0.22 μm的滤膜过滤,即得待测样品溶液。

### 1.3 色谱条件

色谱柱:Hypercarb^TM^多孔石墨碳柱(100 mm ×4.6 mm, 5 μm;美国Thermo公司);柱温:30 ℃;流动相:(A) H_2_O和(B) 25 mmol/L MSA;梯度洗脱程序:0~2.0 min, 2%B; 2.0~2.1 min, 2%B~40%B; 2.1~8.0 min, 40%B; 8.0~8.1 min, 40%B~2%B; 8.1~15.0 min, 2%B;流速:1.0 mL/min;进样量:25.0 μL;检测波长:190 nm。

## 2 结果与讨论

### 2.1 色谱条件的优化

#### 2.1.1 色谱柱和检测波长的选择

依据文献[16,22]的色谱条件,利用C18柱对尿素进行测定,在实际分析中发现,高浓度的 $N{O_{3}}^{-}$ 与尿素难以分离,干扰现象严重,测定结果见图1。

**图1:**
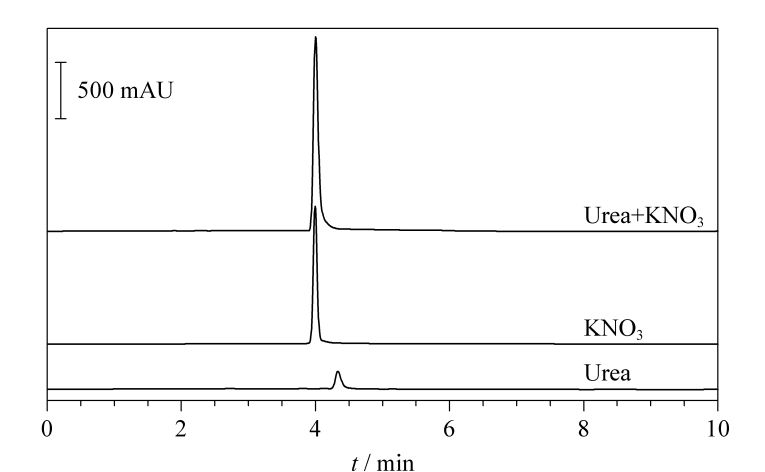
尿素、硝酸钾及其混合溶液在C18柱上的色谱图

因此我们尝试选择Hypercarb柱,发现 $N{O_{3}}^{-}$ 会受到PGC较强的保留作用,使得 $N{O_{3}}^{-}$ 出峰延后,远离尿素峰。这可能是由于 $N{O_{3}}^{-}$ 所带孤对电子与PGC表面产生电子间相互作用,在酸性条件下,阴离子的保留性增强,而在碱性条件下反之。

在对比了尿素以及其他相关杂质离子的紫外光谱图后发现,尿素紫外吸收小,而待测样品中尿素含量很低,在190 nm这一极端波长下,尿素才有较大的响应,满足灵敏度的要求,而在195 nm或更高波长下,难以检测到微量尿素,灵敏度有所欠缺。同时,在190 nm低波长的极端检测条件下,PGC柱的背景噪声相较于C18柱等传统色谱柱而言更小,可在尿素检测时减少背景干扰,因此选择190 nm作为检测波长(通常情况下不采用195 nm及其以下的波长作为紫外检测波长)。

#### 2.1.2 流动相的选择

鉴于尿素在碱性环境下不稳定且易分解,需在中性或酸性流动相中进行分离。选择磷酸、硫酸以及甲基磺酸3种常见的酸性流动相,在相同色谱条件下,分别考察10 mmol/L磷酸、硫酸和5 mmol/L甲基磺酸下的分离效果,见图2。结果表明,磷酸洗脱得到的尿素峰值响应较低,且噪声较大,影响检测灵敏度;而以硫酸为流动相时,在尿素保留时间附近出现倒峰,干扰尿素测定。采用甲基磺酸溶液进行洗脱时,可得到基线平稳、峰形优良的尿素液相色谱图。故最终选择甲基磺酸溶液作为流动相。

**图2:**
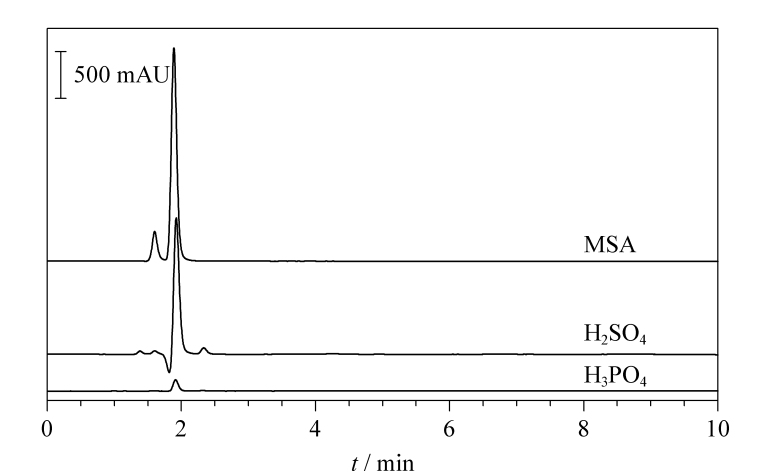
3种酸性流动相下尿素标准溶液的色谱图

#### 2.1.3 流速和柱温的优化

流动相的流速以及色谱柱的柱温均会对待测物的出峰时间以及峰宽造成影响,流速的提高使得柱压增大,从而导致出峰提前;而柱温的改变也会影响待测物与固定相之间的相互作用,发生出峰提前或延后的现象。为确立最佳流速及柱温,在PGC柱上,以5 mmol/L甲基磺酸作为流动相,对所配制的50 mg/L尿素标样进样,首先考察了不同流速(0.3~1.2 mL/min)对分离效果的影响,结果(表1)表明,随着流速增加,尿素保留时间缩短,且峰宽变窄;当流速为1.0 mL/min时,有效塔板数较大,分离效果好。其次考察了不同柱温(20~40 ℃)对检测结果的影响,数据(表2)显示,柱温升高会导致保留时间略有前移,30 ℃下的有效塔板数最高。综合考虑保留时间、塔板数以及分析效率、峰形等因素,最终确定流速为1.0 mL/min,柱温为30 ℃。

**表1:** 不同流速下尿素标样的色谱数据

Flow rate/(mL/min)	t_R_/min	Noise/mAU	N_eff_
0.3	6.167	0.061	2643
0.4	4.643	0.043	2487
0.5	3.727	0.071	2678
0.6	3.109	0.048	2441
0.7	2.673	0.072	2167
0.8	2.350	0.043	2131
0.9	2.095	0.070	2140
1.0	1.890	0.063	2471
1.1	1.717	0.062	2448
1.2	1.580	0.043	2048

*N*_eff_: number of effective plate, plate/m.

**表2:** 不同柱温下尿素标样的色谱数据

Temperature/ ℃	t_R_/min	Noise/mAU	N_eff_
20	1.963	0.072	2375
25	1.921	0.051	2328
30	1.888	0.070	2381
35	1.854	0.043	2192
40	1.826	0.063	2145

#### 2.1.4 浓度优化及干扰离子实验

在流动相洗脱浓度优化实验中,由于样品中除尿素外还含有大量 $N{O_{3}}^{-}$ 、 $N{O_{2}}^{-}$ 、 $N{H_{4}}^{+}$ 、HCOO^-^、 $HC{O_{3}}^{-}$ 等杂质离子,在选择流动相浓度时需考虑尿素与杂质的分离度。本研究初步尝试等度洗脱的方法。分别选取10、5、2.5 mmol/L 3个浓度的甲基磺酸溶液作为流动相进行洗脱。检测结果表明,当流动相浓度不断降低时,尿素保留时间未有明显变化,而 $N{O_{3}}^{-}$ 等离子的保留时间后移。当甲基磺酸浓度为2.5 mmol/L时, $N{O_{3}}^{-}$ 的出峰时间延长至20 min,拉长了检测时间,影响检测效率。而以5 mmol/L甲基磺酸溶液洗脱时, $\;N{O_{2}}^{-}$ 和尿素的色谱峰产生部分重叠(图3a),影响尿素分析的准确性。

**图3:**
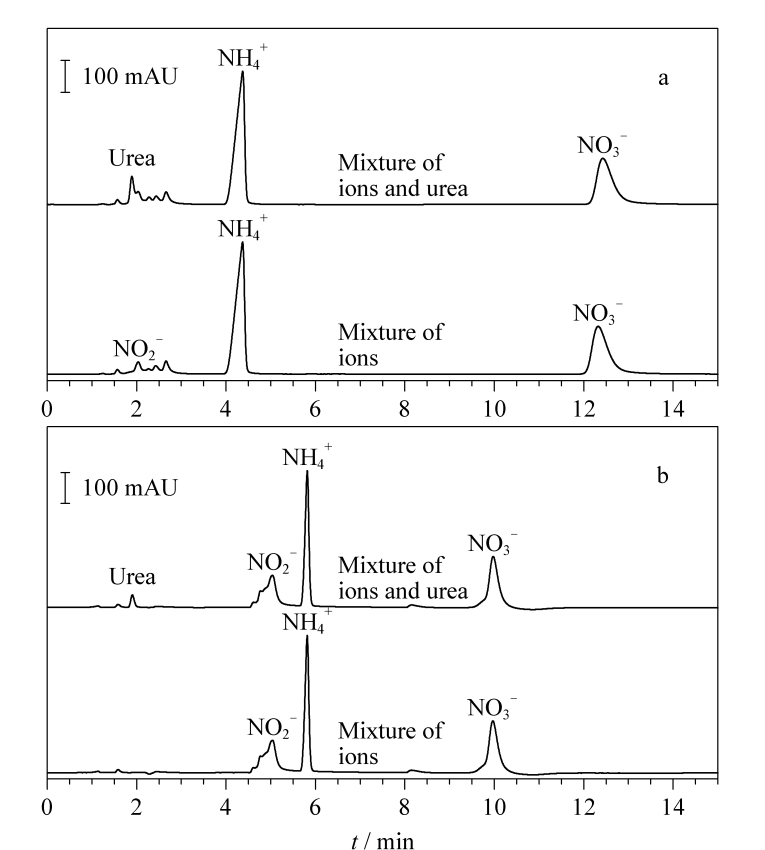
尿素、离子混合标样及离子混合标样的色谱图

鉴于此,为实现尿素与其他离子的完全分离,提高分析效率,进一步开发梯度洗脱的方法。最终确定洗脱程序见1.3节。在此梯度洗脱条件下,对杂质离子混合标样以及添加了尿素的杂质离子混合标样分别进样分析。结果表明,尿素的检测未受到 $N{O_{2}}^{-}$ 等其他杂质离子的干扰(图3b),峰形窄而尖,有利于定性定量分析,且分析时间仅15 min,提高了检测效率。

### 2.2 方法学考察

#### 2.2.1 线性关系、检出限与定量限

根据1.3节所述色谱条件进行测定。待仪器稳定后,对配制好的0.1、1.0、5.0、10、25、50、100 mg/L系列电解液尿素标准溶液依次进样分析,每个水平重复测定3次。分别以峰面积和峰高的平均值为纵坐标,尿素质量浓度为横坐标绘制标准曲线,可得到对应的线性回归方程。结果表明,峰面积和峰高与质量浓度均呈现良好的线性关系,相关系数*r*^2^分别为0.9997和0.9988。同时取噪声0.098 mAU,以*S/N*=3计算方法的检出限,为0.028 mg/L,以*S/N*=10计算方法的定量限,为0.093 mg/L。

#### 2.2.2 重复性

为考察方法的重复性,同样在最佳色谱条件下,对配制好的10、50、100 mg/L 3个质量浓度的标准溶液样品分别连续进样分析,每个水平平行测定8次,记录相应的峰面积和峰高。结果见表3,保留时间及峰面积、峰高的相对标准偏差(RSD)为0.065%~0.79%,说明重复性良好。

**表3:** 3个水平下尿素保留时间、峰面积及峰高的RSD(*n*=8)

Mass concentration/ (mg/L)	Retention time	Peak area	Peak height
10	0.17	0.27	0.79
50	0.081	0.065	0.64
100	0.072	0.084	0.67

#### 2.2.3 加标回收率

通过对实际电解液样品进行加标回收试验考察方法的准确度。选取一个预处理后的0.1 mol/L电解液体系下实际电合成尿素的样品溶液为基底,加入电解液标准样品,得到位于线性范围内的低、中、高3个水平的待测样品,平行测定6次,根据结果计算回收率和RSD值,具体见表4。所得加标回收率为112.0%~118.4%, RSD为3.1%~4.6%,表明该方法准确度良好,能满足实际样品的基本检测需求。

**表 4:** 电解液中尿素的加标回收率和RSD(*n*=6)

Background/ (mg/L)	Added/ (mg/L)	Detected/ (mg/L)	Recovery/ %	RSD/ %
0.126	0.080	0.221	118.2	4.6
	0.10	0.238	112.0	3.1
	0.12	0.268	118.4	4.6

### 2.3 实际样品检测

采用该分析体系,对在0.1 mol/L硝酸钾和碳酸钾电解液体系下进行电化学合成尿素得到的4个实际样品进行检测。利用1.9 min处的尿素峰面积进行定量计算,可得其中一实际样品中的尿素含量为0.126 mg/L,并且通过对比实际样品加标前后的色谱图(图4a和4b),可知1.9 min处的小峰确实为尿素峰,其中图4a为未加标色谱图,图4b为加标100%,即加标0.10 mg/L尿素后的色谱图。图4a和4b在1.5 min左右均出现一明显色谱峰,在对照了干扰离子实验的结果后难以对其进行归属,猜测其主要为在190 nm波长下具有较高响应的杂质,且在实际电合成尿素过程中产生的其他杂质也在死时间位置出峰,进而导致杂质峰更为明显。同时 $N{O_{2}}^{-}$ 以及高浓度的 $N{O_{3}}^{-}$ 等杂质离子的保留时间与标样存在差异,可能是实际样品中的其他未知化合物对其在PGC柱上的保留造成了影响,这些色谱峰并未对低浓度的尿素测定造成干扰。通过峰面积定量计算其余样品中尿素含量分别为0.0963、0.148、0.152 mg/L。说明将该方法应用于实际电合成生产尿素的产物检测是可行的。

**图4:**
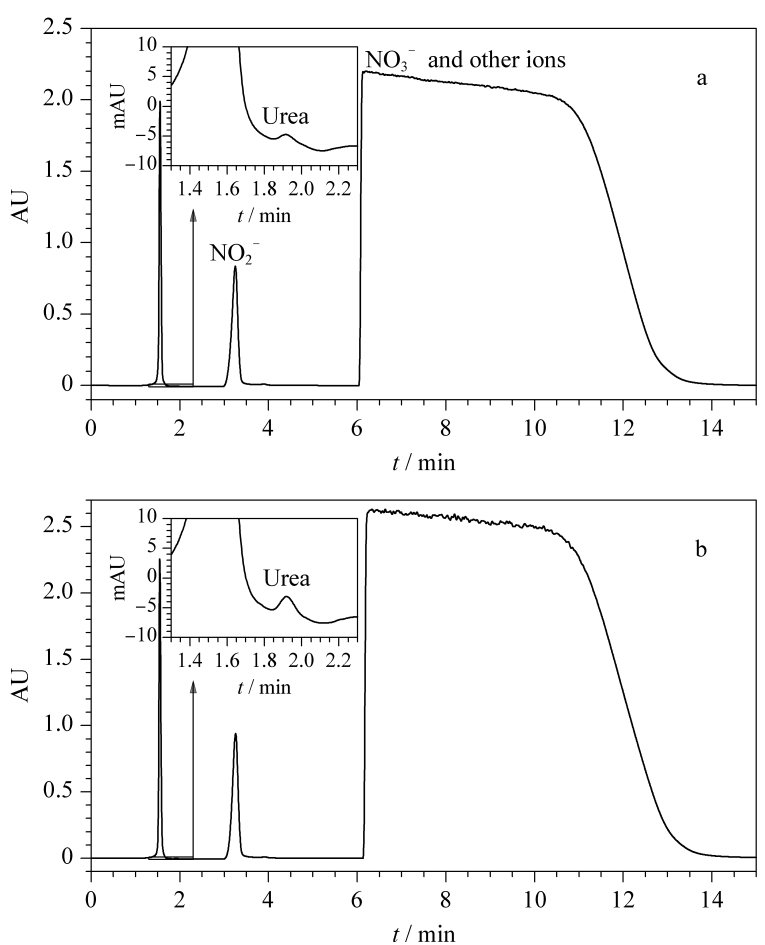
(a)电合成尿素实际样品及其(b)加标样品色谱图

## 3 结论

本研究建立了将PGC柱应用于HPLC以测定电解液体系下微量尿素的分析方法,PGC柱特殊的保留效应能将尿素与其他高浓度干扰离子轻易分开,避免了高浓度基体的干扰,在190 nm的超低检测波长下,既提高了尿素的灵敏度,又保证了较低的背景噪声和基线的稳定性。该方法灵敏度高,准确性好,且前处理步骤简单,检测效率高,为电合成尿素的测定提供了一种新的分析手段。
